# A Rapid Screen for Host-Encoded miRNAs with Inhibitory Effects against Ebola Virus Using a Transcription- and Replication-Competent Virus-Like Particle System

**DOI:** 10.3390/ijms19051488

**Published:** 2018-05-16

**Authors:** Zhongyi Wang, Jiaming Li, Yingying Fu, Zongzheng Zhao, Chunmao Zhang, Nan Li, Jingjing Li, Hongliang Cheng, Xiaojun Jin, Bing Lu, Zhendong Guo, Jun Qian, Linna Liu

**Affiliations:** Academy of Military Medical Sciences, No. 27 Taiping Road, Beijing 100850, China; 15010172111@163.com (Z.W.); ljm35850164@sina.com (J.L.); fyyinging@163.com (Y.F.); zzzfusheng@163.com (Z.Z.); jk704715@sina.com (C.Z.); linan226@126.com (N.L.); lijjamms85@126.com (J.L.); cheng107315@163.com (H.C.); jxj0529@126.com (X.J.); 13693506666@163.com (B.L.); guozd@foxmail.com (Z.G.); qianj1970@126.com (J.Q.)

**Keywords:** Ebola virus, microRNA, inhibition, interaction, trVLPs, BSL-2

## Abstract

MicroRNAs (miRNAs) may become efficient antiviral agents against the Ebola virus (EBOV) targeting viral genomic RNAs or transcripts. We previously conducted a genome-wide search for differentially expressed miRNAs during viral replication and transcription. In this study, we established a rapid screen for miRNAs with inhibitory effects against EBOV using a tetracistronic transcription- and replication-competent virus-like particle (trVLP) system. This system uses a minigenome comprising an EBOV leader region, luciferase reporter, VP40, GP, VP24, EBOV trailer region, and three noncoding regions from the EBOV genome and can be used to model the life cycle of EBOV under biosafety level (BSL) 2 conditions. Informatic analysis was performed to select up-regulated miRNAs targeting the coding regions of the minigenome with the highest binding energy to perform inhibitory effect screening. Among these miRNAs, miR-150-3p had the most significant inhibitory effect. Reverse transcription polymerase chain reaction (RT-PCR), Western blot, and double fluorescence reporter experiments demonstrated that miR-150-3p inhibited the reproduction of trVLPs via the regulation of GP and VP40 expression by directly targeting the coding regions of GP and VP40. This novel, rapid, and convenient screening method will efficiently facilitate the exploration of miRNAs against EBOV under BSL-2 conditions.

## 1. Introduction

MicroRNAs (miRNAs) are efficient antiviral agents [1,2], and the interactions between host-encoded miRNAs and viruses during the processes of viral replication and transcription are of interest [3,4]. The cytotoxicity of the Ebola virus (EBOV) glycoprotein can be reduced by inhibiting Hsa-miR-1246, hsa-miR-320a, and hsa-miR-196b-5p [5], and several miRNAs have been identified as potential pathogen-specific diagnostic biomarkers in circulating microRNA profiles of EBOV infection in both nonhuman primates (NHPs) and humans [6,7]. Additional anti-Ebola miRNAs and their target genes have been predicted in silico [8].

EBOV-related experiments must be performed under biosafety level (BSL) 4 conditions [9], in contrast to the BSL 2 conditions employed in many research experiments. A transcription- and replication-competent virus-like particle (trVLP) system suitable for BSL 2 conditions has been used to study EBOV biology including morphogenesis, budding, entry, genome replication, and transcription. This system employs a tetracistronic minigenome, and its viral components are solely derived from EBOV [10,11]. A rapid screening assay of effective EBOV polymerase inhibitors based on the trVLP system has been performed, and the anti-Ebola effects were confirmed using fully infectious EBOV, confirming the potential utility of this system for identifying anti-Ebola agents [12,13].

In this study, we performed a global screen for anti-Ebola miRNAs and identified their inhibitory effects using a tetracistronic trVLP system that could model the life cycle of EBOV in 293T cells. The miRNA expression data were obtained from our previous study [11]. The target genes of selected up-regulated miRNAs in the EBOV genome were predicted. Among the seven miRNAs examined, three showed a reduction in trVLP titers at 72 h post infection. Particularly, when compared with the other miRNAs targeting VP24 or other noncoding regions, we preferred to explore the mechanisms associated with miRNAs that could potentially target the EBOV GP and VP40 that were of significance to viral infection, replication, packaging, budding, and pathogenicity [14,15,16,17,18]. GP is the transmembrane protein including two subunits—GP1 and GP2—that are connected via a disulfide bond. GP1 contains the receptor binding domain, which can bind to the viral receptor when the virus invades the host cell. GP2 can mediate the fusion process between the virus and cellular membrane [14]. Furthermore, GP can affect the vascular permeability and is associated with the hemorrhagic symptoms of Ebola infection [15,16]. Viral matrix protein VP40 contains many hydrophobic domains and is the viral protein with the highest content, which is about 40% of the whole viral proteins [17,18]. The matrix protein can not only be used as a link bundle between the viral envelope and internal nucleic acids, but can also play an important role in viral packaging and budding [17]. The host-encoded miRNAs with inhibitory effects against GP and VP40 may significantly decrease the packaging efficiency of viral particles and reduce the viral pathogenicity induced by GP. Then we selected miR-150-3p and its associated target genes to verify the interactions between them, and the results showed that miR-150-3p exclusively inhibited viral GP and VP40 protein production.

## 2. Results

**Figure 1:**
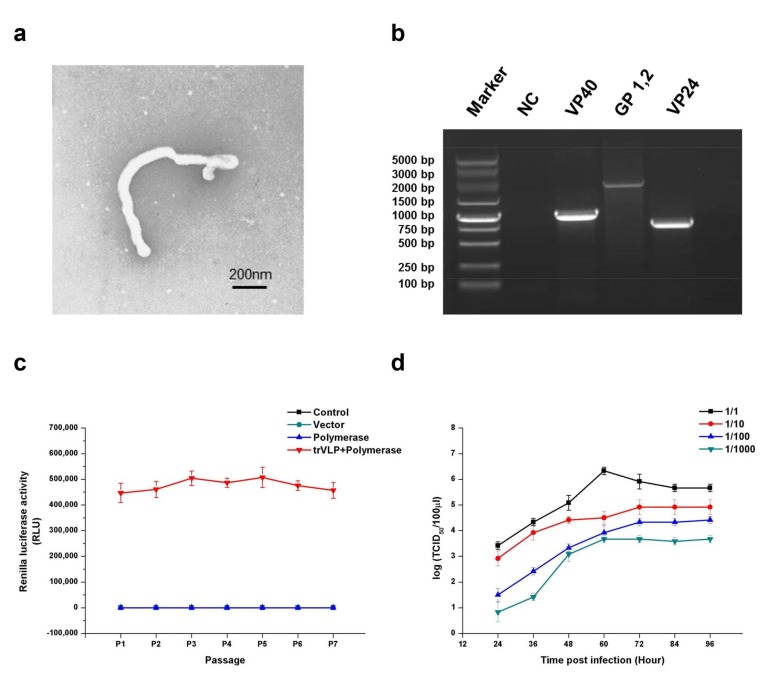
Morphological, genetic, and stability identification; stability analysis; and proliferative ability determination of transcription- and replication-competent virus-like particles (trVLPs). (**a**) Electron microscopy of trVLPs. P1 trVLPs were concentrated using centrifugal filter devices (Millipore Corporation, Temecula, CA, USA) and then observed by electron microscopy. Scale is 200 nm. (**b**) Genetic identification of VP40, GP, and VP24. Reverse transcription polymerase chain reaction (RT-PCR) was performed to obtain the complete sequences of the three genes in the minigenome. The products of RT-PCR were identified by sequencing. NC: Cells with no trVLP infection. (**c**) Renilla luciferase activity from P1 to P7. To evaluate its hereditary stability, the trVLPs containing the recombinant virus genome were carried on to successive generations, and the Renilla luciferase activities from P1 to P7 were detected. In particular, the Renilla luciferase activities of the three groups (control, vector, and polymerase) were almost at the same level. The products of RT-PCR for the trVLP genome from P0 to P7 were identified by sequencing. The data of this sequencing experiment confirmed that the sequences of the trVLP genome were the same as those of wild-type EBOV (Zaire Mayinga Ebola virus) and the sequences remained unchanged from P0 to P7. (**d**) Proliferation curve of trVLPs in 293T cells. The virus proliferation curve in 293T cells was constructed by detecting the 50% tissue culture infective dose (TCID_50_) of the trVLPs from the cell culture supernatant. The TCID_50_ of trVLPs used for infection were 10^−6.5^/mL, 10^−5.5^/mL, 10^−4.5^/mL, and 10^−3.5^/mL from 1/1 to 1/1000, accordingly. The results in (**c**,**d**) are presented as the mean ± SD of three independent experiments.

First, morphological and genetic identifications were performed to ensure that the trVLPs produced in this study could be used to explore EBOV biology and the antiviral effects of miRNAs targeting viral gene expression. Electron microscopy confirmed that the filamentous form, radius, and length of these trVLPs were similar to those of true EBOV (Figure 1a). Reverse transcription polymerase chain reaction (RT-PCR) and sequencing experiments confirmed that the sequences of GP, VP40, and VP24 were the same as those of wild-type EBOV (Zaire Mayinga Ebola virus) (Figure 1b). To evaluate its heredity stability, the trVLPs containing the recombinant virus genome were carried on to successive generations. The products of RT-PCR for the trVLP genome from P0 to P7 were identified by sequencing. The data of this sequencing experiment confirmed that the sequences of trVLP genome remained unchanged from P0 to P7. The Renilla luciferase activity remained relatively stable from P1 to P7 (Figure 1c). We previously determined the 50% tissue culture infective dose (TCID_50_) of the trVLPs by measuring Renilla activity [11]. Here, we constructed the virus multiplication curve in 293T cells by detecting the TCID_50_ of the trVLPs from the cell culture supernatant (Figure 1d). The results of these analyses confirmed that the trVLPs produced in this study could be used to explore the inhibitory effects of potential anti-Ebola miRNAs.

**Figure 2:**
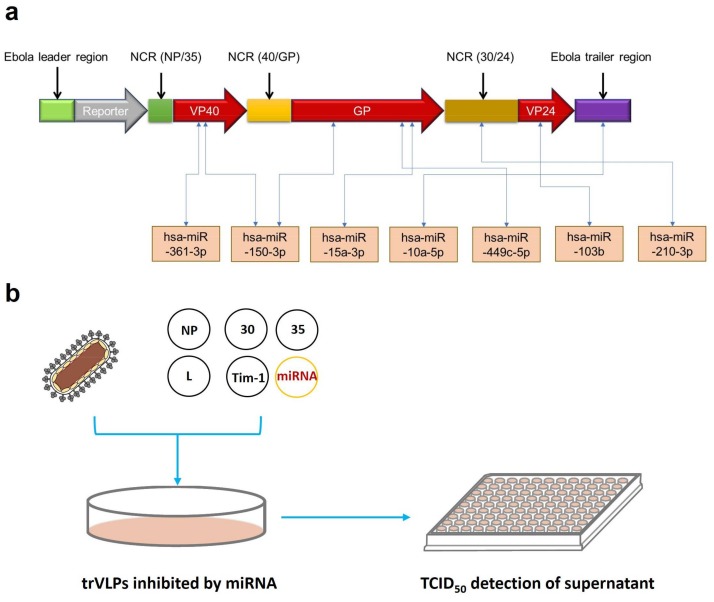
Target prediction and processes of screening for anti-Ebola microRNAs (miRNAs). (**a**) Potential target prediction of miRNAs. Interactions between mRNAs and miRNAs were predicted by the online tool RNAhybrid. We selected the seven miRNAs with the highest binding energy for rapid screening for anti-Ebola miRNAs. (**b**) The whole process of preliminary screening for miRNAs with inhibitory effects. The screening process mainly comprised miRNA expression by plasmids, trVLP infection, and TCID_50_ detection of the trVLPs in cell culture supernatants.

The tetracistronic minigenome of the trVLPs contains the EBOV leader region, luciferase reporter, VP40, GP, VP24, Ebola virus trailer region, and three noncoding regions from the EBOV genome that are required to produce trVLPs containing these minigenomes [10]. Here, we predicted the binding sites of up-regulated miRNAs selected from our previous transcriptional analysis [11] targeting VP40, GP, VP24, and other regions in the transcripts from this minigenome. The interactions of mRNAs with miRNAs were predicted by using RNAhybrid [19]. The information details of seven selected miRNAs with the highest binding energy are shown in Table 1 with the RNA sequence and minimum free energy (MFE). For example, hsa-miR-150-3p had the highest binding energy, with −33.6 kcal/mol for GP and −32.0 kcal/mol for VP40. Then, the selected miRNAs targeting different regions of the minigenome were used to continue the next experiments (Figure 2a). P1 trVLPs were used to infect 293T cells that were pretransfected with pCAGGS-L, pCAGGS-NP, pCAGGS-VP30, pCAGGS-VP35, pCAGGS-Tim1, and pGV251 or pGV514, each expressing miRNAs. The TCID_50_ of P1 trVLPs was 10^−3.5^/mL. Approximately 2 × 10^5^ cells were present in each well of a 12-well plate, and 1 mL of P1 trVLPs was added per well. The cell supernatants were collected 72 h post infection, and their TCID_50_ values were determined as previously described (Figure 2b). 

**Figure 3:**
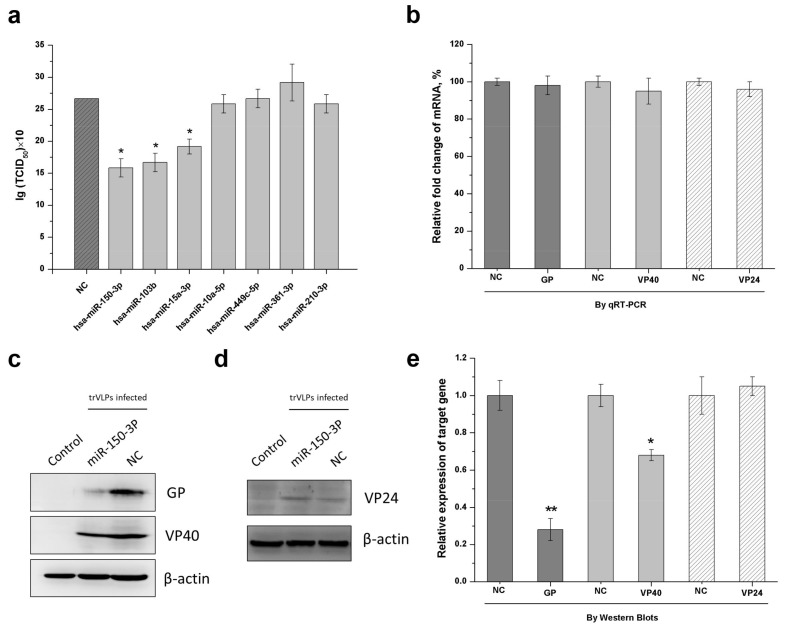
miR-150-3p inhibits the reproduction of trVLPs by inhibiting GP and VP40 expression. 293T cells were transfected with support plasmids and a miRNA-expressing plasmid, and then infected with trVLPs. (**a**) Detection of the inhibitory effect from each miRNA is shown in Table 1. The best inhibitory effect against trVLP was achieved by miR-150-3p, as determined by the TCID_50_ of the cell culture supernatant 72 h post infection. NC: empty vector was used as a control. (**b**) At 72 h post infection, the total RNA was extracted, reverse-transcribed, and quantified by qPCR. Relative fold changes in mRNA were compared with infected, non-miR150-3p-expressing cells (NC). Charcoal grey represents the mRNA fold change of GP; light grey represents the mRNA fold change of VP40; white with slashes represents the mRNA fold change of VP24. The protein levels of GP, VP40 (**c**), and VP24 (**d**) were analyzed by Western blotting. β-actin was used as an internal reference. Control: cells with no infection; NC: cells with infection; miR-150-3p: infected cells with the expression of miR-150-3p. (**e**) The intensities of the respective images in (**c**,**d**) were quantified using ImageJ software. Charcoal grey represents the relative expression of GP; light grey represents the relative expression of VP40; white with slashes represents the relative expression of VP24. The results in (**a**,**b**,**e**) are presented as the mean ± SD of three independent experiments; * *p* < 0.05; ** *p* < 0.01.

**Figure 4:**
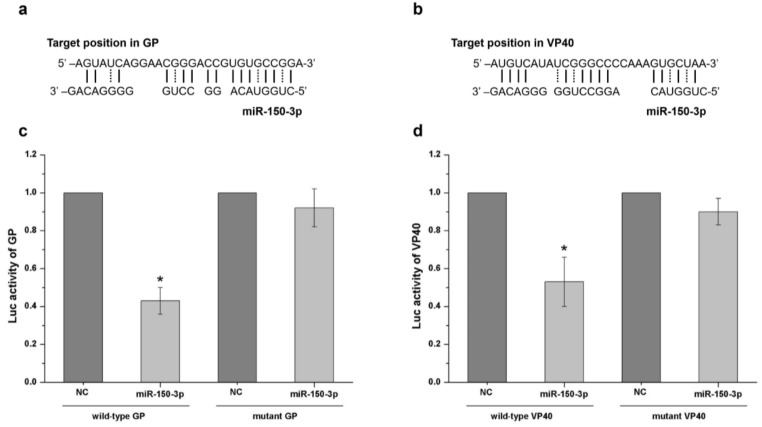
Confirmation that miR-150-3p targets GP and VP40. Subfigures (**a**,**b**) show that miR-150-3p matched the potential binding sites of GP and VP40 (indicated by solid and dotted lines). The interactions of mRNAs with miRNAs were predicted by miRNA target prediction software such as the TargetScan and Bielefeld services online. The miRNA binding site on GP transcript: position 420–447; the miRNA binding site on VP40 transcript: position 239–265. (**c**) Wild-type and mutant GP binding site activity analysis by the luciferase reporter test. The vector expressing miR-150-3p was co-transfected with the vector containing the wild-type or mutant binding region. NC represents cells with no miR-150-3p expression. (**d**) Wild-type and mutant VP40 binding site activity analysis by the luciferase reporter test. The vector expressing miR-150-3p was co-transfected with the vector containing the wild-type or mutant binding region. NC represents cells with no miR-150-3p expression. The results are presented as the mean ± SD of three independent experiments; * *p* < 0.05.

As shown in Figure 3a, the screening results revealed that three miRNAs—miR-150-3p, miR-103b, and miR-15a-3p—had inhibitory effects, as demonstrated by a significant decrease in TCID_50_. Among these miRNAs, miR-150-3p had the most obvious inhibitory effect and targeted both the GP and VP40 transcripts. Furthermore, when compared with other miRNAs, we preferred to explore the mechanisms associated with miR-150-3p because miR-150-3p could potentially target the EBOV GP and VP40 that are of significance to viral infection, replication, packaging, budding, and pathogenicity. This miRNA was therefore selected to verify its inhibition mechanism and the associated interaction details. RT-PCR and Western blotting were performed to clarify whether the inhibitory effect occurred at the transcription or translation level. The details of the RT-PCR method were described in a previous study [12]. For the Western blots, we used polyclonal antibodies against EBOV-GP and EBOV-VP40 produced in horse and polyclonal antibodies against EBOV-VP24 produced in mice [20]. There was no significant difference in the relative fold change of the mRNAs of GP, VP40, and VP24 between the infected, miR-150-3p-expressing cells and infected, non-miR-150-3p-expressing cells (Figure 3b). However, the expression of GP and VP40 in the infected, miR-150-3p-expressing cells was significantly reduced, while the expression of VP24 was the same between these two types of cells (Figure 3c–e). As shown in Figure 3b–d, the expression of GP and VP40 was unchanged at the transcriptional level, but decreased at the protein level in the miR-150-3p group. Informatic analysis revealed that miR-150-3p matched the coding regions of GP and VP40 (Figure 4a,b). We then constructed an miR-Report luciferase reporter to determine whether miR-150-3p directly targeted the coding regions of GP and VP40. The miR-Report luciferase reporter was co-transfected with the miR-150-3p expression plasmid. The luciferase activity of the reporter containing the wild-type coding region for GP or VP40 was suppressed by miR-150-3p, whereas the luciferase activity of the reporter containing a mutant coding region was not affected (Figure 4c,d).

## 3. Discussion

MiRNAs could play important roles in many human diseases [21,22,23,24,25] and these miRNA-based cellular responses to viral infection might provide a balance between virus reproduction and normal cellular mechanisms [26,27,28]. However, host miRNAs that target the Ebola genome with an inhibitory effect against EBOV reproduction have been rarely reported. In fact, many experiments related to EBOV need BSL-4 lab conditions, which might increase the study cost and limit the depth of research [29]. In this study, we established a novel platform that could be used as an efficient and rapid tool to screen miRNAs with an inhibitory effect against EBOV reproduction under BSL-2 lab conditions using a trVLP system. We performed a series of experiments to identify the characteristics of the trVLP system including the morphological features, genetic stability, and proliferative ability (Figure 1). The results confirmed that this system models the life cycle of EBOV and represents an efficient tool for exploring antiviral agents during the processes of viral replication, transcription, and translation.

EBOV infection can induce many differentially expressed host-encoded miRNAs [11,30], which might provide antiviral effects by directly or indirectly targeting viral replication, transcription, and translation. As there was a balance between viral reproduction and cellular responses [3], the increase in expressed miRNAs following viral reproduction might play an important role against viral replication and transcription processes [31,32]. To find the miRNAs targeting the EBOV genome that could be considered for the application of effective therapeutic strategies against Ebola hemorrhagic fever, informatic analysis was performed to select up-regulated miRNAs targeting the coding regions of the minigenome with the highest binding energy for inhibitory effect screening (Figure 2). The potential direct binding between miRNAs and mRNAs might influence the gene expression at a translational level. Herein, we found that miR-150-3p, miR-103b, and miR-15a-3p had the best inhibitory effect. Then, we performed a series of experiments to explore the interaction mechanisms between the miRNAs and trVLPs, which might explain the antiviral effects of these cellular miRNAs. The results of RT-PCR, Western blot, and double-luciferase reporter experiments (Figure 3 and Figure 4) indicated that miR-150-3p inhibits the reproduction of trVLPs via the regulation of GP and VP40 expression by directly targeting the coding regions of GP and VP40. As shown in Figure 3a, only miR-150-3p, miR-103b, and miR-15a-3p had an inhibitory effect on trVLP reproduction and the other four miRNAs did not show any significant reduction in trVLP titers. This different functional phenomenon of different miRNAs might be due to their different target binding ability as miR-10a-5p, miR-449c-5p, miR-361-3p, and miR-210-3p had lower binding scores in the bioinformatic prediction analysis.

Although studies have shown that host miRNAs can regulate viral infection by directly targeting viral genomic RNAs or the transcripts relevant to their replication, miRNAs can also be responsible for both adaptive immune response and innate antiviral immune responses [3]. Furthermore, some cellular metabolisms may also be regulated by host miRNAs, which may contribute to the antiviral effect following expression of highly specific miRNAs [22,33]. In this study, we found that miR-150-3p could suppress the expression of GP and VP40, which could contribute to the inhibitory effect against trVLP reproduction as GP and VP40 are of importance to viral infection, replication, and pathogenicity. However, we cannot rule out some of the other indirect roles of miR-150-3p that could further affect viral reproduction. Future studies may find other interesting functions provided by miE-150-3p that directly or indirectly target EBOV and these potential findings may further improve upon the conclusions in this study.

Unlike some anti-EBOV antibody agents that directly target the viral proteins, host miRNAs could regulate the viral protein expression [33]. This function may reduce not only the virus titers, but also the cytotoxicity of the viral proteins to host cells [5,34]. Besides this, miRNAs could be much less expensive and easier for us to produce and deliver to humans than traditional antibody agents. Due to the rapid genetic evolutionary rates of the Ebola virus, the miRNAs with species specificity should be more suitable for the prevention and control of EBOV by choosing constantly updated miRNAs that perfectly match the changing sequences of the EBOV genome [35]. All in all, the experimental design and screening strategy proposed here will provide guidance for future anti-EBOV studies based on miRNAs or other agents targeting the processes of EBOV replication, transcription, and translation.

## 4. Materials and Methods

### 4.1. Cell Culture and trVLPs Preparation

HEK-293T cell lines were maintained in Dulbecco’s modified Eagle medium (Corning Corporation, New York, NY, USA) containing 8% fetal bovine serum (Corning Corporation) and 1% penicillin/streptomycin (Gibco, Grand Island, NY, USA) in a humidified atmosphere with 5% CO_2_ at 37 °C. Five plasmids named pCAGGS-L, pCAGGS-NP, pCAGGS-VP30, pCAGGS-VP35, and pCAGGS-T7 were used for the P0 trVLP production, and pCAGGS-L, pCAGGS-NP, pCAGGS-VP30, pCAGGS-VP35, and pCAGGS-Tim1 were used to prepare target cells. The production details of P1 trVLP have been described in a reference paper [10,11]. The TCID_50_ of P1 trVLPs for the evaluation of the miRNA inhibitory effect was 10^−3.5^/mL.

### 4.2. Luciferase Assay for trVLPs Reproduction

Renilla luciferase was monitored using a Renilla-Glo Assay Kit (Promega, Madison, WI, USA). A total of 40 μL of each sample was added to 40 μL of Renilla-Glo reagent, and the mixture was then measured in a luminometer using an integration time of 1 s [10,11]. 

### 4.3. TCID_50_ Assay

Ninety-six-well tissue culture plates were used to perform a TCID_50_ assay in this study. Cell culture supernatants of different test groups containing trVLPs were diluted into different concentrations. The technical details of cell culture and reporter activity detection have been described in previous studies [10,11]. Any well with an RLU three times higher than the control group was identified as positive. Then, the TCID_50_ was calculated by the Karber method [36].

### 4.4. Western Blot Analysis

The protein concentration of lysed cell samples was determined by using a bicinchoninic acid protein assay kit (Thermo Fisher Scientific Corporation, Shanghai, China). Proteins were separated by sodium dodecyl sulfate polyacrylamide gel electrophoresis (10%) and were electro-transferred to a nitrocellulose blotting membrane (GE Healthcare Life Science, Shanghai, China) using a Trans-Blot SD Semi-Dry Transfer Cell (Bio-Rad Corporation, Hercules, CA, USA) for 25 min at room temperature. Blots were incubated overnight at 4 °C with antibodies against EBOV-GP and EBOV-VP40 (1:2000 dilution; horse antibody, provided by Professor Yang Songtao) [20], followed by incubation for 1 h at room temperature with a horseradish-peroxidase-conjugated rabbit anti-horse secondary antibody (Bioss Corporation, Beijing, China). The primary antibody for VP24 was the mouse polyclonal antibody (produced and stored in our laboratory). The primary antibody for internal control was mouse β-actin monoclonal antibody (Santa Cruz Biotechnology Corporation, Dallas, TX, USA). The secondary antibody for VP24 and β-actin detection was peroxidase-conjugated goat anti-mouse IgG antibody (Biosharp Corporation, Beijing, China). All the images were obtained from Chemiluminescence (Millipore Corporation).

### 4.5. Real-Time PCR Quantification of Viral RNA

Total RNA was extracted from cells using TRIzol reagent (Invitrogen Corporation, Carlsbad, CA, USA). RT-PCR was performed to evaluate trVLP hereditary stability and to clarify whether the inhibitory effect occurred at the transcription or translation level. The details of the specific RT-PCR method have been described in a previous study [12]. GP forward_1 (5′-ATG GGC GTT ACA GGA ATA TTG-3′), GP reverse_1 (5′-CTA AAA GAC AAA TTT GCA TAT ACA G-3′), VP40 forward_1 (5′-ATG AGG CGG GTT ATA TTG CCT ACT-3′), VP40 reverse_1 (5′-TTA CTT CTC AAT CAC AGC TGG AAG AC-3′), VP24 forward_1 (5′-ATG GCT AAA GCT ACG GGA CGA TAC-3′) and VP24 reverse_1 (5′-TTA GAT AGC AAG AGA GCT ATT AAA TTC AAG-3′) were used in this study for gene sequence detection in Figure 2b. GP forward_2 (5′-TGG GCT GAA AAC TGC TAC AAT C-3′), GP reverse_2 (5′-CTT TGT GCA CAT ACC GGC AC-3′), VP40 forward_2 (5′-CCT ACT GCT CCT CCT GAA T-3′), VP40 reverse_2 (5′-TTG CTG TTG CCA CCT CTA-3′), VP24 forward_2 (5′-TAA CAA CCA ACA CTA ACC-3′) and VP24 reverse_2 (5′-AAT ACT GCT CAA CAA TCC-3′) were used for qRT-PCR quantification in Figure 3b.

### 4.6. Plasmid Construction and Dual-Luciferase Activity Assay

The plasmids expressing miRNAs were constructed by Genechem Corporation in China based on the vector pGV514 and pGV251 and the related methods were described in previous studies [37,38]. The plasmids expressing miRNAs, which were treated by transient transfection, could remain stable from 0 to 72 h post trVLP infection with the same observation result of GFP under fluorescence microscope [37,38]. As for the miR-150-3p, its stability in cell culture has been confirmed in previous studies [39,40], which means it can provide sustained functions of affecting protein synthesis post trVLP infection. As shown in Figure 2b, all the plasmid transfection was done 24 h before trVLP infection. The amount of transfected plasmids expressing miRNAs was 1 µg in each well of a 12-well plate. The amounts of pCAGGS-NP, pCAGGS-VP35, pCAGGS-VP30, pCAGGS-L, and pCAGGS-Tim1 were 70 ng, 70 ng, 30 ng, 500 ng, and 125 ng in each well of a 12-well plate, accordingly. The process of transfection work was the same as a previous study described [10,11].

The fragments of GP and VP40 containing miR-150-3p binding sites or the corresponding mutated binding sites were synthesized and cloned into the luciferase vector pGV272. The fragment of GP cloned into the vector was position 339–546. The fragment of VP40 cloned into the vector was position 159–364. Cells were transfected with wild-type or mutated constructs in the presence of miR-150-3p-expressing plasmid or its control by using Lipofectamine 2000 (Invitrogen). Luciferase activity was measured using the Dual-Luciferase Reporter Assay System (Promega) according to the manufacturer’s instructions. Renilla luciferase activity was normalized to the activity of Firefly luciferase. 

## Figures and Tables

**Table 1:** Information of selected microRNAs. The MFE (minimum free energy) of each microRNA was predicted using the RNAhybrid Service.

MicroRNA Name	Sequence	MFE (kcal/mol)
hsa-miR-150-3p	ctggtacaggcctgggggacag	−33.6 for GP or −32.0 for VP40
hsa-miR-15a-3p	caggccatattgtgctgcctca	−31.4 for GP
hsa-miR-449c-5p	taggcagtgtattgctagcggctgt	−31.2 for Reporter
hsa-miR-10a-5p	taccctgtagatccgaatttgtg	−31.1 for Ebola trailer region
hsa-miR-361-3p	tcccccaggtgtgattctgattt	−30.5 for VP40
hsa-miR-210-3p	ctgtgcgtgtgacagcggctga	−30.0 for NCR (30/24)
hsa-miR-103b	tcatagccctgtacaatgctgct	−29.3 for VP24
